# Genomic and Pathologic Characterization of the First FAdV-C Serotype 4 Isolate from Black-Necked Crane

**DOI:** 10.3390/v15081653

**Published:** 2023-07-29

**Authors:** Xiaoyan Xue, Qinhong Yang, Ming J. Wu, Zhenxing Zhang, Jianling Song, Wei Wang, Jia Yang, Jia Ji, Yongxian Zhang, Hongyang Dai, Hongbin Yin, Suhua Li

**Affiliations:** 1 College of Life Sciences, Southwest Forestry University, 300 Bailong Road, Kunming 650024, China; xuexiaoyan@swfu.edu.cn (X.X.); yang-qinhong@swfu.edu.cn (Q.Y.); 2031wei@swfu.edu.cn (W.W.); yangjia0806@hotmail.com (J.Y.); lisuar@163.com (J.J.); 2 School of Science, Western Sydney University, Locked Bag 1797, Penrith, NSW 2751, Australia; m.wu@westernsydney.edu.au; 3 Yunnan Tropical and Subtropical Animal Virus Diseases Laboratory, Yunnan Academy of Animal Husbandry and Veterinary Sciences, 6 Qinglongshan, Kunming 650224, China; zhenxing978@163.com; 4 Animal Disease Inspection and Supervision Institution of Yunnan Province, 118 Gulou Road, Kunming 650051, China; tyh2001x@outlook.com (Y.Z.); freshyhb@hotmail.com (H.Y.); 5 The Management Bureau of Huize Black Necked Crane National Nature Reserve, 744 Tongbao Road, Qujing 654200, China; w15398745109@hotmail.com

**Keywords:** *Fowl aviadenovirus C* (FAdV-C), fowl adenovirus serotype 4 (FAdV-4), phylogenic analysis, pathologic analysis, mallard duck, black-necked crane

## Abstract

Fowl adenoviruses (FAdVs) are distributed worldwide in poultry and incriminated as the etiological agents for several health problems in fowls, and are capable of crossing species barriers between domestic and wild fowls. An FAdV strain was, for the first time, isolated from black-necked crane in this study, and was designated as serotype 4 *Fowl aviadenovirus C* (abbreviated as BNC2021) according to the phylogenetic analysis of its DNA polymerase and *hexon* gene. The viral genomic sequence analysis demonstrated that the isolate possessed the ORF deletions that are present in FAdV4 strains circulating in poultry fowls in China and the amino acid mutations associated with viral pathogenicity in the hexon and fiber 2 proteins. A viral challenge experiment with mallard ducks demonstrated systemic viral infection and horizontal transmission. BNC2021 induced the typical clinical signs of hepatitis–hydropericardium syndrome (HHS) with swelling and inflammation in multiple organs and showed significant viral replication in all eight organs tested in the virus-inoculated ducks and their contactees at 6 dpi. The findings highlight the importance of surveillance of FAdVs in wild birds.

## 1. Introduction

The family of Adenoviridae was divided into six genera, *Mastadenovirus*, *Siadenovirus*, *Atadenovirus*, *Ichtadenovirus*, *Testadenovirus* and *Aviadenovirus,* in the 2022 ICTV (International Committee on Taxonomy of Viruses) report [1]. Fowl adenoviruses (FAdVs), belonging to the genus *Aviadenovirus*, have been prevalent worldwide in poultry and incriminated as the etiological agents for a number of clinical conditions in fowls such as inclusion body hepatitis (IBH), hepatitis–hydropericardium syndrome (HHS), adenoviral gizzard erosion (AGE) and gizzard erosion (GE) [2,3]. Based on genomic differences and serum cross-neutralization tests of the viral hexon protein, FAdVs are classified into five species, *Fowl aviadenovirus A* to *Fowl aviadenovirus E* (FAdV-A to FAdV-E), with a total of 12 serotypes (FAdV-1 to FAdV-8a and FAdV-8b to FAdV-11) [1,4].

The circulation of FAdVs in China can be traced back to 1976, when the first IBH case in poultry was reported in mainland China [5]. FAdV-4, FAdV-8a, FAdV-8b and FAdV-11 were isolated from diagnostic materials of the sporadic or cluster distribution of IBH and HHS cases in some regions of China from 2007 to 2014, with FAdV-11 being the predominant serotype [5]. FAdV-4 emerged as the most pathogenic causative agent responsible for the outbreaks of HHS characterized by high mortality and rapid epidemic spread in China between 2015 and 2018 [6]. In a recent epidemiological survey in 2019, all 12 serotypes were detected in apparently healthy fowl species (chicken, goose, duck, pigeon and partridge) throughout China, with FAdV-1, FAdV-2, FAdV-3 and FAdV-4 showing relatively high prevalence [7]. The diverse range of FAdV species presents significant challenges for the prevention and control efforts.

FAdVs in the genus *Aviadenovirus* have frequently been detected in various wild bird species globally [8]. While the host range of adenoviruses has been generally confined to single or evolutionarily closely related species, numerous adenoviruses can cross the host species barriers and infect new organisms. For instance, the FAdV-D and FAdV-E strains were identified in diseased falcons with IBH syndrome and they shared low identity (∼35%) with *Falcon adenovirus* (FaAdV), another member of the genus *Aviadenovirus* [9]. 

Considering the prevalence of FAdVs in domestic poultry in China, and their potential for cross-species transmission, we conducted a surveillance of FAdVs in wild birds as part of a comprehensive virus surveillance program on wildfowls in Yunnan Province, China, initiated by the local administrative department of wildlife conservation [10]. Specifically, a strain of FAdV-4 was successfully isolated from a fecal sample of black-necked crane (*Grus nigricollis*). Complete viral genome sequences were determined and characterized and its pathogenicity on domestic mallard ducks was evaluated. These findings may contribute valuable data on the prevalence of FAdVs in wild birds and the potential risk they pose to domestic poultry and wild fowls.

## 2. Materials and Methods

### 2.1. Fecal Samples from Wading Birds

A total of 814 fecal samples were collected from wading birds in the natural reserves in Yunnan Province, China. Detailed information regarding sample collection was given in a previous study [10].

### 2.2. PCR Protocols for Virus Identification

The viral DNA was extracted using a QIAamp viral RNA/DNA kit (Qiagen, Hilden, Germany) according to the manufacturer’s instructions. The reported degenerate primers of hexon A (5′-CAARTTCAGRCAGACGGT-3′) and hexon B (5′-TAGTGATGMCGSGACATCAT-3′), which bind to the two pedestal regions adjacent to the L1 loop region of the *hexon* gene of the FAdV-A-1 strain CELO (with the GenBank accession number of U46933), were employed to amplify the 12 serotypes of FAdVs to detect the presence of FAdVs [11]. FAdV-positive samples were further subjected to virus isolation through propagation in the Leghorn Male Hepatoma (LMH, ATCC, Manassas, USA) cell line. Subsequently, the lysates of LMH cells underwent a second PCR process using the reported primers (forward primer 5′-ATACCAACACGAGCACCAC-3′ and reverse primer 5′-TTATCCCTGAACCCGATG-3′) specific for the *hexon* gene of the FAdV-4 strain KR5 (with the GenBank accession number of HE608152.1) [12]. The selection of FAdV-4 primers for the second PCR step was based on the results of subsequent next-generation sequencing.

### 2.3. Virus Isolation Using LMH and Determination of TCID_50_

LMH suspension cells were cultured in Dulbecco’s modified Eagle’s medium (DMEM) (Gibco, Grand Island, NY, USA) at a cell density of about 3 × 10^5^ cells/mL in a humidified atmosphere with 5% CO_2_ at 37 °C. These cells were then infected with FAdV-positive fecal samples. The fecal samples were aseptically filtered through 0.22 μm membranes prior to the cell inoculation. The cytopathic effect (CPE) in LMH cells was monitored daily, and the viral infectivity of the median tissue culture infectious dose (TCID_50_) was determined as described previously [13,14]. Briefly, serial dilutions (10^−7^ to 10^0^) of cell lysates from the previous generation were inoculated into LMH cells in 96-well plates with 12 repeats per dilution. Infected units refers to the wells exhibiting cytopathic effects (CPE), while non-infected units were wells without CPE. The cumulative number of infected units was calculated by summing the infected units at each dilution, assuming that infections persisted across lower dilutions and higher viral titers. Similarly, the cumulative number of non-infected units were determined based on the assumption that units remain uninfected at higher dilutions if they were not infected at a certain dilution. TCID_50_ was obtained from cumulative infected and non-infected units using the Reed–Muench method [14].

### 2.4. Determination of Complete Genome

To obtain complete viral genomes, lysates of FAdV-propagated LMH cells were subjected to next-generation sequencing (NGS). The viral DNA library was generated using a Nextera XT DNA Library Preparation Kit (Illumina, San Diego, USA) and paired-end sequencing was performed on the Illumina HiSeq 2 500 platform (Illumina, CA, USA). A screening against the *Gallus gallus*-5.0 genome (with the GenBank accession number of No. GCA_000002315.5) and the mitochondrial genome of *Gallus sonneratii* (with the GenBank accession number of No. AP006746.1) was employed to eliminate carrier genome interference. After filtering unqualified data using Fastx version 0.0.13 online software (http://hannonlab.cshl.edu/fastx_toolkit/index.html accessed on 21 April 2023), the remaining unmapped reads were labeled as clean reads for subsequent analysis. Contigs assembled from these clean reads via SPAdes version 3.5.0 software (CAB SPbU, St Petersburg, Russia) were aligned through Accelrys Gene version 2.5 (Accelrys, CA, USA) with manual sorting and orienting. Finally, annotations were performed by comparing sequences against the complete FAdV genomes available in the GenBank database.

### 2.5. Sequence Aligment Analysis

The genomic sequences of five FAdV species (FAdV-A to FAdV-E) and other viral species in *Aviadenovirus* were retrieved from the GenBank database (Supplementary Table S1). These sequences were aligned with the isolate in this study using the online MAFFT (Multiple Alignment using Fast Fourier Transform) tool (https://mafft.cbrc.jp/alignment/software/ accessed on 21 April 2023). Maximum likelihood (ML) phylogenetic trees were constructed based on the amino acid sequences of DNA polymerase and nucleotide sequences of the *hexon* gene, respectively. IQ-TREE version 2.0.3 was implemented for tree construction [15], utilizing the substitution models of LG+F+I+G4 (for DNA polymerase) and TN+F+R 3 (for *hexon*) with 1 000 bootstrap replicates. The developed trees were visualized using FigTree version 1.4.4 (http://tree.bio.ed.ac.uk/software/figtree/ accessed on 21 April 2023).

Open reading frames (ORFs) were predicted using the online NCBI ORF Finder tool (https://www.ncbi.nlm.nih.gov/orffinder/ accessed on 21 April 2023) with a minimum length of 50 codons. The deduced protein sequences from the predicted ORFs were compared against a non-redundant protein database using the BLAST (Basic Local Alignment Search Tool) program. Hits matching the existing ORFs in other FAdVs were considered as correct ORFs. The predicted ORFs were visualized using the GSDS web server (Gene Structure Display Server, http://gsds.gao-lab.org accessed on 21 April 2023) for drawing gene structure schematic diagrams [16]. Amino acid site mutations associated with viral pathogenicity in fiber 2 and hexon proteins were analyzed and compared between other representative FAdV-4 strains.

### 2.6. Viral Challenge Study with Mallard Ducks

One-day-old mallard ducks (*Anas platyrhynchos*) purchased from a commercial duck farm in Yiliang City, Yunnan Province, China, were provided with ad libitum feed and drink and maintained at a temperature of 30 °C on the first day, gradually decreasing by 0.5 °C every day until 20 °C was reached, with a relative humidity if 40–60% and a 22: 2-h light–dark cycle. All the ducks used in this study were unimmunized and tested negative for FAdV through serological and etiological analysis. Prior to virus inoculation, serum samples were collected for ELISA testing using a commercial detection kit (BioChek, Bodegraven, The Netherlands) and oropharyngeal and cloacal swab samples were collected for PCR testing.

Thirty-six 3-week-old mallard ducks, identified with numbered anklets, were randomly assigned to three groups: inoculation group, contact group and control group, each consisting of twelve ducks. The ducks in the inoculation group were intramuscularly injected with 1.0 mL of the isolated strain in the lysates of LMH cells at a dose of 10^6.17^ TCID_50_/200 μL. The ducks in the contact and control groups received equivalent volumes of normal DMEM cell culture medium without virus via the intramuscular route. The ducks in the inoculation and contact groups were co-housed, while those in the control group were housed separately. Over a period of 14 days, the ducks were continuously monitored for changes in body weights and clinical signs. At 3, 6, 10 and 14 days post inoculation (dpi), three ducks from each group were euthanized using carbon dioxide, and the organs, including heart, liver, spleen, lung, kidney, thymus, duodenum and bursa of Fabricius, were collected for gross lesion observation, histopathologic examination and viral load quantification.

### 2.7. Histopathologic Examination

At 6 dpi, tissues from the inoculation and control groups were fixed in 10% formalin, dehydrated with graded alcohol, cleared with xylene and embedded in paraffin. Subsequently, 5 μm sections from the paraffin-embedded tissues were deposited onto glass slides. Hematoxylin–eosin (HE) staining was performed on the tissue sections to visualize histopathologic changes under a microscope. Images were captured using an Olympus CX31 microscope (Olympus, Tokyo, Japan) and imported using the CCD imaging system (Motic, Xiamen, China).

### 2.8. Quantification of Viral Titers in Tissues

To assess the replication of the isolated strain in mallard ducks, viral titers were quantified in eight different tissues. Firstly, PCR products from the amplification of the FAdV-4 *hexon* gene were cloned into pMD-18T vectors and transformed into *E.coli* DH5α competent cells. The resulting plasmid, pMD-18T-FAdV-4, was validated through Sanger dideoxy sequencing. Then, viral DNA samples with concentrations ranging from 10^0^ copies/μL to 10^6^ copies/μL were generated from the plasmid through 10-fold gradient dilution, and they served as the templates of a TaqMan-based qualitative real-time PCR (qPCR) protocol using TaKaRa probe qPCR Mix with UNG (TaKaRa, Kusatsu, Japan), as previously described [17]. The qPCR analysis employed specific primers (FAdV-4-F: 5′-CGTCAACTTCAAGTACTC-3′, FAdV-4-R: 5′-AGAGGATGCTCATGTTAC-3′ and a TaqMan probe 5′-FAM-CCTACTCAGATGGAGGCTTCTACC-TAMRA-3′). Next, a standard curve was generated by plotting the logarithm of viral DNA copies against the obtained cycle threshold (CT) values. Each tissue sample, weighing 100 mg, was homogenized in 1 mL PBS. Viral DNA was extracted from 200 μL supernatant of each sample, followed by TaqMan-based qPCR. Finally, CT values were recorded and converted into the viral DNA copies using the standard curve.

### 2.9. Statistical Analysis

The viral titers in different tissue samples from mallard ducks acquired at 3, 6, 10 and 14 dpi were statistically analyzed through two-way ANOVA, using the GraphPad Prism software version 9.5.0 (GraphPad, San Diego, USA). A *p* value < 0.05 was considered statistically significant.

## 3. Results

### 3.1. Identification of the Isolated Strain BNC2021

**Figure 1:**
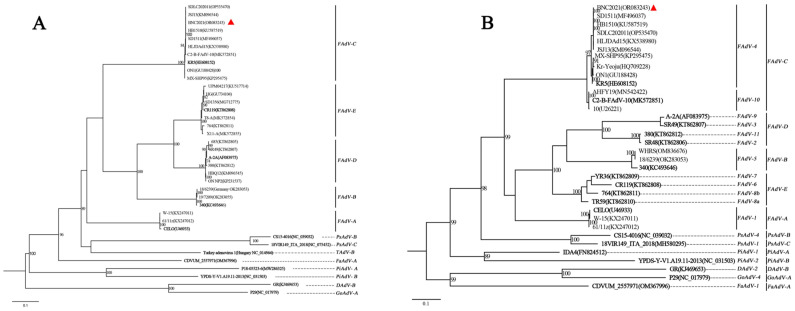
Maximum likelihood phylogenetic analysis based on the amino acid sequences of DNA polymerase and nucleotide sequence of *hexon*. BNC2021, denoted by a red triangle, is the FAdV-4 strain isolated in this study. The official reference strains are presented in bold in each branch. (**A**) In the DNA polymerase amino acid tree, BNC2021 is clustered with the official references of the *Fowl aviadenovirus C* (FAdV-C) strain KR5. (**B**) The *Hexon* tree classifies the 12 serotypes of fowl aviadenovirus (FAdV) and BNC2021 clusters with the serotype 4 FAdV (FAdV-4). Branch supports are assessed using 1000 bootstrap replicates, and only values higher than 70% are displayed at the branch nodes.

One fecal sample from the black-necked crane was confirmed to be positive for FAdV through PCR using primers of *hexon* A and *hexon* B. It was classified as FAdV-C and FAdV-4 according to the phylogenetic analysis of DNA polymerase and *hexon*, using the official reference strains from previous studies and the GenBank Database [1,18] (Figure 1). The strain was proven to be FAdV-4 through PCR protocol with the specific primers of the FAdV-4 *hexon* gene. This novel strain was designated as FAdV-C serotype 4/black-necked crane/YNZT/BNC/2021 and abbreviated as BNC2021. Its genomic sequences were deposited in GenBank (with accession number of OR083243).

BNC2021 showed a similarity of over 99% to the FAdV-4 strains that circulated in poultry in China, such as HLJDAd15 (with the GenBank accession number of KX538980), SDLC202011 (with the GenBank accession number of OP535470), JSJ13 (with the GenBank accession number of KM096544), SD1511 (with the GenBank accession number of MF496037) and HB1510 (with the GenBank accession number of KU587519) (Figure 1). In the phylogenetic tree of DNA polymerase, BNC2021 clustered with the FAdV-C strains, exhibiting a distance matrix of 0.0036 from KR5 (with the GenBank accession number of HE608152), the official reference strain of FAdV-C. This suggested that BNC2021 belonged to FAdV-C, based on the standard for species designation of adenoviruses (Figure 1A). The phylogenetic tree for *hexon* classified the 12 serotypes of FAdVs, positioning BNC2021 within the topology cluster of FAdV-4 (Figure 1B). Therefore, BNC2021 from black-necked crane was identified as FAdV-4.

### 3.2. Genomic Analysis of BNC2021

**Figure 2:**
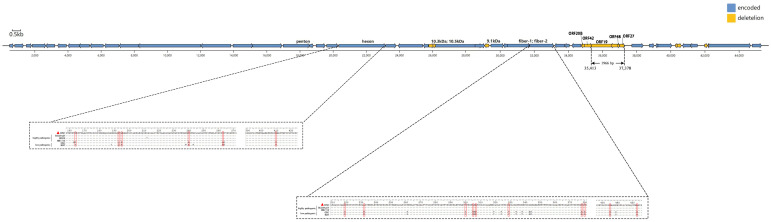
Schematic diagram of fowl adenovirus (FAdV) genome and the mutations in the fiber 2 and hexon proteins of fowl adenovirus serotype 4 (FAdV-4) strains. BNC2021, denoted by a red triangle, is the strain isolated in this study. The position and direction of transcription for the predicted open reading frames (ORFs) are indicated by blue arrows, while deletions of ORFs in BNC2021 are represented by yellow arrows. The red boxes highlight the amino acid sequence mutations in fiber 2 and hexon. The FAdV-4 strains marked here include highly pathogenic strains circulating in China (HLJDAd15, SDSX and HB1510), as well as representatives of low-pathogenic strains (ON1 and KR5).

Genomes of the BNC2021 strain totaled 43 710 bp, containing 43 ORFs (Figure 2 and Supplementary Table S1). Certain ORFs, including ORF19, 10.3 kDa, 10.5 kDa, ORF20B, ORF48 and ORF27, which were displayed in some low-pathogenic strains such as ON1 (with GenBank access number GU188428) and KR5, were absent in BNC2021. Notably, the missing sequences of ORF19, ORF27 and ORF48 contributed to a 1966 bp deletion at the right arm end. This deletion pattern has been commonly observed in circulating FAdV-4 strains in China in recent years.

Fiber 2 has binding sites for the cellular receptor and hexon exhibits a variability of the L1 loop region. Both play crucial roles in the pathogenicity of FAdV-4. Mutations in the L1 loop region of hexon (T^164^S, Q^193^R, E^195^Q, A^240^T, M^263^I and T^410^A) and in the fiber 2 protein (G^219^D, E^232^Q, I^300^ T, S^305^ A, P^307^ A, V^329^ L, A^378^ T, A^380^ T, T^435^ S, T^453^ A and S^453^A) might be involved in the viral infectivity (Figure 2).

### 3.3. Pathogenicity Assessment of BNC2021 in Mallard Ducks

LMH cells inoculated with BNC2021 exhibited shrinking in size, adopting a round in shape, and aggregated in grape-like clusters commencing from the third passage. The TCID_50_ for BNC2021 on LMH was determined to be 10^6.17^ at 60 h post inoculation. This TCID_50_ was subsequently used in the viral challenge study involving mallard ducks.

**Figure 3:**
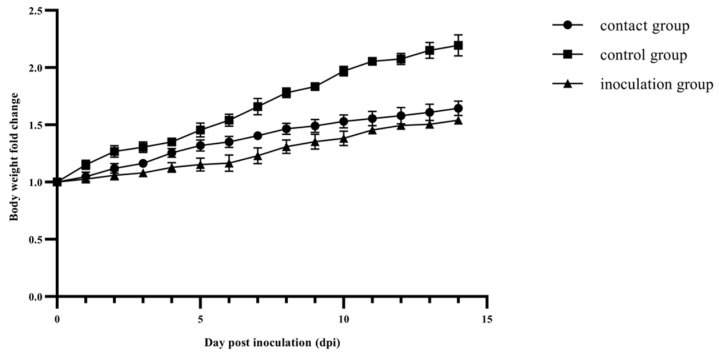
The changes in body weight of mallard ducks over a period of 14 days post inoculation (dpi). Body weights are graphed as the fold change, comparing them to the initial values on the day of inoculation (0 dpi). The inoculation group consisted of 3-week-old mallard ducks intramuscularly inoculated with BNC2021. The contact and control groups are composed of ducks inoculated with normal DMEM cell culture medium without virus via the same route. The ducks in the inoculation and contact groups were co-housed, while the control group was housed separately.

Ducks in the inoculation and contact groups exhibited mild clinical symptoms, such as ruffled feathers, anorexia and depression at 2 to 4 dpi, as well as the occasional loose green stools at 6 dpi. Furthermore, consistent body weight gains were observed in the control group. In contrast, the inoculation group exhibited suppressed body weight gain from 1 to 6 dpi, while the contact group experienced the suppression at 8 to 10 dpi. Specifically, at 6 and 8 dpi, the control group exhibited a 1.54- and 1.78-fold increase, respectively, compared to the original weight on the day of inoculation. In comparison, the body weight gain for the inoculation group was only a 1.17-fold change at 6 dpi and a 1.31-fold for the contact group at 8 dpi (Figure 3).

**Figure 4:**
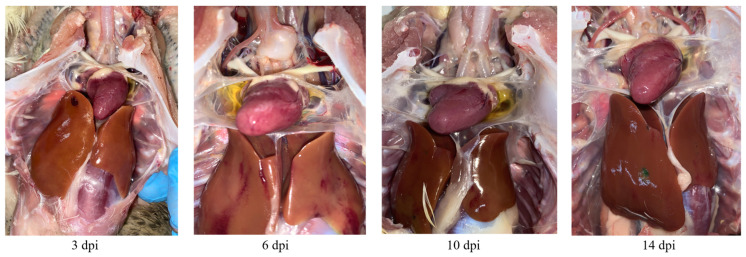
Pathological fluid accumulation in the pericardial sac observed at 3, 6, 10 and 14 days post inoculation (dpi) in ducks from the inoculation group.

The individuals from the BNC2021 inoculation group exhibited typical signs of HHS, including earth-yellow liver discoloration and hemorrhaging, and accumulations of straw-colored fluid in the pericardial sac. Macroscopically, pathological fluid accumulation in the ducks from the inoculation group was detected at 3 dpi, reached its maximum at 6 dpi and then subsided at 14 dpi (Figure 4). Additionally, swelling, soft texture or hyperemia were observed in the heart, liver, spleen, lung, kidney, thymus and bursa of Fabricius of the ducks in the inoculation group. 

**Figure 5:**
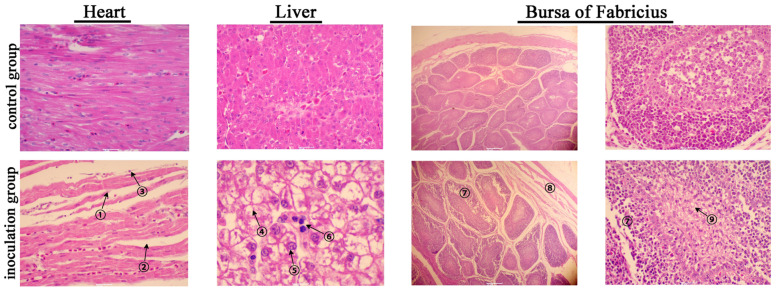
Histopathological analysis through HE staining of paraffin-embedded tissue slices from mallard ducks infected with BNC2021. No lesions are observed in the control group. The lesions in the contact group are similar to those in the inoculation group, and are therefore not shown. Microscopic lesions in heart are characterized by myocardial fiber fracture ①, myocardial interstitial edema ② and inflammatory lymphocyte invasion ③. Microscopic liver lesions are characterized by hydropic degeneration ④, necrosis ⑤ and lymphocyte infiltration ⑥. Microscopic lesions in the bursa of Fabricius are characterized by interstitial edema in the lymphocytes of the cortex ⑦ and tunica muscularis ⑧, as well as lymphocytic degeneration or necrosis ⑨. Bar = 20 μm.

Histological HE-stained sections from the heart, liver and bursa of Fabricius at 6 dpi are shown in Figure 5. In the heart, myocardial fibers displayed fragmentation and curling at the edges. The enlargement and increased water content of the intercellular spaces were observed due to myocardial interstitial edema. Additionally, lymphocyte infiltration was evident in the myocardial interstitium, indicating an inflammatory response had been triggered. Hepatocytes displayed hydropic degeneration characterized by a honeycomb appearance with varying sizes of vacuoles within the cytoplasm. Some cells exhibited necrosis, which could be identified by the dissolution of the nucleus with a loss of structural integrity and staining intensity. It was also accompanied by inflammatory lymphocyte infiltration. Notably, the basophilic inclusion bodies in the hepatocyte nucleus were absent in this case, which were transient and sometimes difficult to identify. The histopathological changes observed in the bursa of Fabricius were mainly located in the bursal follicles. In the outer cortex, clearly visible intercellular spaces were present among lymphocytes that should be densely packed, indicating the presence of interstitial edema. In the inner medulla, lymphocytes showed varying changes in ballooning degeneration or necrosis with the disappearance of nuclei. Interstitial edema was also observed in the tunica muscularis (Figure 5).

### 3.4. Viral Titers in Tissues from Mallard Ducks Challenged with BNC2021

**Figure 6:**
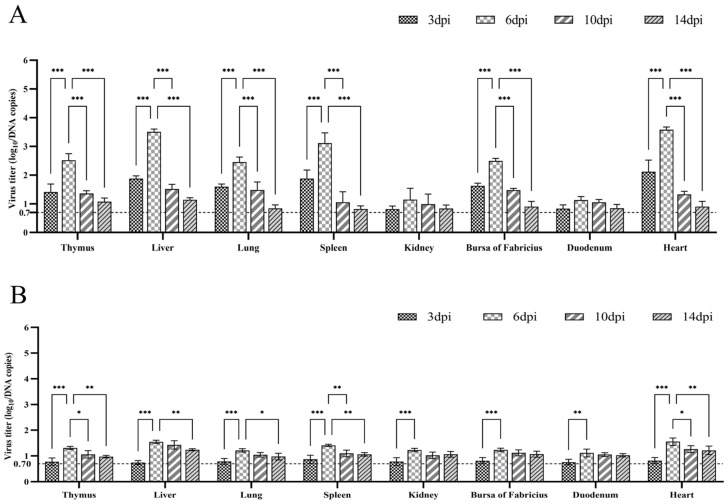
Virus titers in various organs of mallard ducks from the inoculation group (**A**) and the contact group (**B**). Three ducks from each group were euthanized at 3, 6, 10 and 14 dpi, and the hearts, livers, spleens, lungs, kidneys, thymuses, duodena, and bursas of Fabricius were collected. Viral loads were generated from the CT (cycle threshold) values of qPCR according to the standard curve. Samples that fell below the detectable levels were assigned a value of 0.70, which is equivalent to the log_10_ value of the detection limit of 5 copies/μL. GraphPad Prism software version 9.5.0 was used to perform two-way ANOVA, and the symbols ***, ** and * indicate significance levels of *p* < 0.001, *p* < 0.01 and *p* < 0.05, respectively.

A standard curve generated with plasmid pMD18T-FAdV-4 was used to convert the CT values from qPCR amplification into infectious units represented by viral DNA copies. The detection limit was 5 copies/μL, with a CT value of 35. If the CT values exceeded 35, viral titers were assigned a value of 0.70, which represented the log_10_ value of DNA copies. The highest viral titers were observed at 6 dpi in the inoculation group, with all three samples from each of the eight organs exhibiting detectable levels. Viral titers in the heart, liver, spleen, lung, thymus and bursa of Fabricius at 6 dpi were significantly higher compared to those at 3 dpi, 10 dpi or 14 dpi (*p* < 0.001; Figure 6A). This observation coincided with the peak pathological fluid accumulation of HHS at 6 dpi. Additionally, viral replication decreased to levels undetectable with qPCR in the majority of the samples at 14 dpi (Figure 6A). BNC2021 viral titers were significantly higher in the heart, liver and spleen tissues compared to the lung, thymus, bursa of Fabricius, kidney and duodenum (*p* < 0.01). Interestingly, in the contact group, approximately two thirds of samples displayed BNC2021 replication at 6, 10 and 14 dpi, while viral titers were only around one to ten percent of those observed in the inoculation group at 6 dpi (Figure 6B). This may suggest the horizontal transmission of BNC2021. 

## 4. Discussion

The phylogenetic distance of DNA polymerase served as an effective approach in the identification of adenovirus species [1]. Additionally, the *hexon* gene, which encodes a major viral surface-exposed capsid structural protein, proved particularly useful for distinguishing FAdV serotypes due to its significant variability [18]. The isolated strain of this study, BNC2021, was identified according to the phylogenetic trees from both the amino acid sequence of DNA polymerase and nucleotide sequence of *hexon* gene (Figure 1). It is most likely the first FAdV-4 isolate from black-necked crane, as no available data can be found in the GenBank database. This study expands the host range of FAdV-4 to include wild bird species of crane. Notably, this identification does not represent the exclusive FAdV-4 strain isolated from wild avian host species. A strain of FAdV-4 from wild black kites in Kashipur, Uttarakhand, India, was identified using agar gel immunodiffusion and immunofluorescence tests with specific serotype antiserum in 2010 [19]. In 2015, three FAdV-4 strains were isolated from ostriches with the clinical signs of HPS in China, based on *hexon* loop 1 gene sequence analysis [5].

Adenoviruses from the genera *Aviadenovirus*, *Atadenovirus* and *Siadenovirus* have been found in wild birds [1,4]. In addition, the vast number of unknown adenoviruses in wild birds remains a significant challenge due to the high divergence of newly discovered avian adenoviruses and the current research biases towards human and domesticated animals [20]. It is likely that there are additional adenovirus species that have not yet been identified in cranes or other wild birds, because the current study only focused on the five FAdV species and their 12 serotypes.

A wintering population of approximately 4300 black-necked cranes, known as the Eastern Population, has been identified in the Yunnan–Guizhou plateaus, China [21]. The Dabaoshan black-necked crane nature reserve, situated in Yunnan Province, is the primary wintering site for the population. Satellite tracking data reveal that these birds migrate from Dashanbao to the breeding site of the Ruoergai marshes, which is located in the northwest of Sichuan Province and houses the largest breeding population of black-necked crane worldwide [22]. This indicates that black-necked cranes migrate within China. Thus, the BNC2021 isolated in this study likely originated from domestic poultry in China and was then transmitted to wild cranes. This inference was supported by the high similarity in genome sequences between BNC2021 and poultry-originated strains found across China, such as SDLC202011, JSJ12, SD1511 and HB1510. Furthermore, the presence of a distinct 1966 bp deletion in BNC2021 (Figure 2), observed exclusively in poultry-originating strains, further supports this conclusion [12,23,24].

FAdVs can be transmitted from domestic poultry to wild birds, and wild birds may also serve as the reservoirs of FAdVs and interspecies transmission may occur the other way around [25,26]. Mallard ducks were selected as experimental animals for this viral challenge study due to their breeding in backyard farms and shared aquatic environments with black-necked cranes. The findings from the pathological examination and viral load detection (Figure 3, Figure 4, Figure 5 and Figure 6) provided confirmation of this two-way viral transmission route. Additionally, the findings reveal that ducks could potentially act as natural hosts for FAdV-4, which had been established in a previous report [27]. In the animal experiment, a small paddling pool was provided to emulate the water environment for farmyard ducks. The ducks in the contact and inoculation groups were co-housed and shared the same water pool. The presence of high viral titers and histopathological changes in the organs of ducks from the contact group indicated BNC2021 infection (Figure 6). The infection likely occurred via horizontal transmission facilitated by the shared water between the two groups.

Field cases of HHS in ducks caused by FAdV-4 demonstrate a high mortality rate [28,29,30,31]. However, mild symptoms of pericardial effusion and hepatitis were induced in mallard ducks by BNC2021 in this study. Consistently, the degeneration and necrosis of parenchymal cells, interstitial edema and inflammatory lymphocyte infiltration were observed, indicating inflammatory response against BNC2021. It is worth mentioning that ducks infected with the FAdV-4 strain HLJDAd15 also exhibited only mild clinical signs [27], and the extensive organ damage with hyperemia and enlargement in the liver were also observed in FAdV-4-infected Jinding ducks [32]. Furthermore, no cranes were found dead from clinical diseases in the sampling period of this study, suggesting BNC2021 was mildly pathogenic to wild cranes and mallard ducks.

Previous studies have demonstrated the significant roles of fiber 2 and hexon in the pathogenicity of FAdV-4, with fiber 2 mediating FAdV infection by binding to its cellular receptor and hexon facilitating virus tissue tropism and pathogenicity through the variability of its L1 loop region [24,33]. The mutations related to high pathogenicity were observed in the fiber 2 and hexon proteins of BNC2021 (Figure 2). Hence, the pathogenicity of BNC2021 in ducks contradicts its molecular feature of pathogenicity-related mutations. One possible explanation could be that the virulence of BNC2021 was attenuated through adaptation during propagation in LMH cells. This type of virulence attenuation has also been reported in the LMH-cell-adapted FAdV-4 strains HLJDAd15 and SDXL, which were isolated from commercial ducks that died from HHS in China, and conversely induced low pathogenicity in the tested experimental ducks [30,34].

Taken collectively, FAdV-4 was, for the first time, isolated from black-necked crane. The isolate (BNC2021) was analyzed through genomic sequencing and phylogenetic alignments. The strain was further characterized through a viral challenge study in mallard ducks. The findings demonstrate that cranes, and possibly other wild bird species, are hosts of FAdV-4. This poses a serious challenge to both the conservation of cranes and the domestic poultry industry. As the birds can migrate across the borders of neighboring countries, screening and surveillance of avian adenoviruses should be coordinated amongst neighboring countries in order to control and prevent viral epidemics.

## Data Availability

Not applicable.

## Supplementary Materials

The following supporting information can be downloaded at: https://www.mdpi.com/article/10.3390/v15081653/s1, Table S1: The detailed information of the sequences of *DNA polymerase* and *hexon* retrieved from the GenBank database used for phylogenetic analysis in this study; Table S2: Orientations, locations and amino acid sizes of predicted ORFs in BNC2021; Figure S1: Gross lesions observed in the organs of mallard ducks infected with BNC2021.
