# Supplementary material for: Genomic and Pathologic Characterization of the First FAdV-C Serotype 4 Isolate from Black-Necked Crane
Source: Viruses. 2023 Jul 29;15(8):1653. doi: 10.3390/v15081653 (PMC10458181; doi:10.3390/v15081653)
Supplement: Supplementary file 1 [file viruses-15-01653-s001.zip › viruses-2476369-supplementary.pdf]

Supplementary Table S1. The detailed information of the sequences of *DNA polymerase* and *Hexon* gene retrieved from GenBank Database used for phylogenetic analysis in this study.

**Virus strains**	**Accession number**	**Species**	**Serotype**
W-15	KX247011	*Fowl aviadenovirus A*	1
61/11z	KX247012	*Fowl aviadenovirus A*	1
CELO	U46933	*Fowl aviadenovirus A*	1
WHRS	OM836676	*Fowl aviadenovirus B*	5
340	KC493646	*Fowl aviadenovirus B*	5
18/6239	OK283053	*Fowl aviadenovirus B*	5
19/7209	OK283055	*Fowl aviadenovirus B*	5
ON1	GU188428	*Fowl aviadenovirus C*	4
MX-SHP95	KP295475	*Fowl aviadenovirus C*	4
KR5	HE608152	*Fowl aviadenovirus C*	4
HLJDAd15	KX538980	*Fowl aviadenovirus C*	4
HB1510	KU587519	*Fowl aviadenovirus C*	4
SDLC202011	OP535470	*Fowl aviadenovirus C*	4
JSJ13	KM096544	*Fowl aviadenovirus C*	4
SD1511	MF496037	*Fowl aviadenovirus C*	4
CH/GDYF/201706	MK387062	*Fowl aviadenovirus C*	4
CH/AHMG/2018	MN606303	*Fowl aviadenovirus C*	4
Kr-Yeoju	HQ709228	*Fowl aviadenovirus C*	4
C2-B-FAdV-10	MK572851	*Fowl aviadenovirus C*	10
10	U26221	*Fowl aviadenovirus C*	10
AHFY19	MN542422	*Fowl aviadenovirus C*	10
685	KT862805	*Fowl aviadenovirus D*	2
SR48	KT8h62806	*Fowl aviadenovirus D*	2
SR49	KT862807	*Fowl aviadenovirus D*	3
A-2A	AF083975	*Fowl aviadenovirus D*	9
380	KT862812	*Fowl aviadenovirus D*	11
HBQ12	KM096545	*Fowl aviadenovirus D*	11
ON NP2	KP231537	*Fowl aviadenovirus D*	11
CR119	KT862808	*Fowl aviadenovirus E*	6
YR36	KT862809	*Fowl aviadenovirus E*	7
X11-A	MK572855	*Fowl aviadenovirus E*	7
T8-A	MK572854	*Fowl aviadenovirus E*	8a
TR59	KT862810	*Fowl aviadenovirus E*	8a
764	KT862811	*Fowl aviadenovirus E*	8b
UPM04217	KU517714	*Fowl aviadenovirus E*	8b
HG	GU734104	*Fowl aviadenovirus E*	8b
SD1356	MG712775	*Fowl aviadenovirus E*	8b
QD2016	MF577036	*Fowl aviadenovirus E*	8b
P29	NC_017979	*Goose aviadenovirus A*	4
P18-05523-6	MW286325	*Pigeon aviadenovirus A*	1
YPDS-Y-V1.A19.11-2013	NC_031503	*Pigeon aviadenovirus B*	2

**Virus strains**	**Accession number**	**Species**	**Serotype**
CS15-4016	NC_039032	*Psittacine aviadenovirus B*	4
18VIR149_ITA_2018	NC_075452	*Psittacine aviadenovirus C*	1
D90/2	NC_014564	*Turkey aviadenovirus B*	1

Supplementary Table S2. Orientations, locations and amino acid sizes of predicted ORFs in the BNC2021.

**Name**	**Direction[1]**	**Location**	**Numbers of amino acid**
ORF0	R	477-716	80
ORF1	R	789-1313	175
ORF1B	R	1484-1807	108
ORF2	R	1849-2667	273
ORF24	L	2669-3205	179
ORF14A	L	3317-3904	196
ORF14	L	3998-4684	229
ORF14B	L	4668-5297	210
ORF13	L	5331-6131	267
ORF12	L	6208-7077	290
IVa2	L	7091-8275	395
DNA pol	L	8259-12014	1252
pTP	L	12 019-13 827	603
52/55kDa	R	13 957-15 159	401
pIIIa	R	15 146-16 918	591
Penton	R	16 990-18 567	526
pVII	R	18 576-18 809	78
pX	R	18 981-19 520	180
pVI	R	19 624-20 307	228
Hexon	R	20 353-23 166	938
Protease	R	23 184-23 813	210
DBP	L	23 934-25 415	494
11.7kDa	L	25 454-25 762	103
100kDa	R	25 794-28 955	1067
22/33kDa	R	28 576-29 160	195
9.1kDa	L	29 104-29 352	83
pVIII	R	29 474-30 217	248
U-exon	L	30 162-30 449	96
Fiber-1	R	30 448-31 743	432
Fiber-2	R	31 727-33 166	480
ORF22	L	33 219-33 809	197
ORF20A	L	33 812-34 078	89
ORF20	L	34 229-35 116	296
ORF42	R	34 955-35 368	138
ORF43	R	35 777-36 445	223
ORF28	R	36 801-37 094	98
ORF29	R	37 169-37 360	64
GAM-1	R	37 342-38 157	272
ORF17	L	38 773-39 249	159
ORF16	L	39 242-39 652	137
Hypothetical	L	40 085-40 231	49
ORF19A	R	42 722-40 230	831

Name	Direction[1]	Location	Numbers of amino acid
ORF4	R	42 816-43 313	166

[1] R for rightward-transcribed strand and L for leftward-transcribed strand

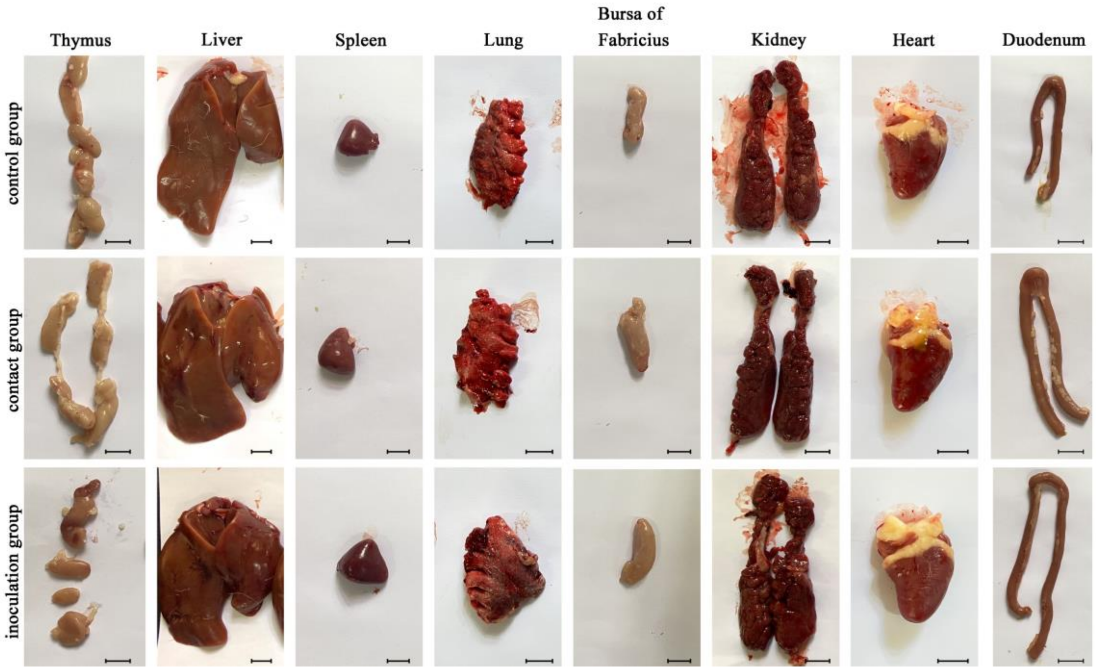

Supplementary Figure S1. Gross lesions observed in the organs of mallard ducks. The inoculation group consisted of 3-week-old mallard ducks intramuscularly inoculated with BNC2021. The contact and control groups are composed of ducks inoculated with normal DMEM cell culture medium without virus via the same route. The ducks in the inoculation and contact groups were co-housed, while the control group was housed separately. At 6 day post inoculation (dpi), hyperemia characteristics were observed in the tissues of thymus, lung, and duodenum of the inoculation and contact groups; swelling was observed in the tissues of liver, spleen, bursa of Fabricius, kidney, and heart; and softening of tissue texture was observed in the tissues of lung, liver, and kidney. Bar=1 cm.
